# COVID-19 Knowledge, Attitudes, and Vaccine Hesitancy in Ethiopia: A Community-Based Cross-Sectional Study

**DOI:** 10.3390/vaccines11040774

**Published:** 2023-03-31

**Authors:** Muluken Dessalegn Muluneh, Kasahun Negash, Sentayehu Tsegaye, Yared Abera, Derbe Tadesse, Sintayehu Abebe, Cathy Vaughan, Virginia Stulz

**Affiliations:** 1 Amref Health Africa in Ethiopia, P.O. Box 20855, Addis Ababa 1000, Ethiopia; 2 Melbourne School of Population and Global Health, Melbourne University, Parkville, VIC 3010, Australia; 3 School of Nursing and Midwifery, Western Sydney University, Sydney, NSW 2751, Australia

**Keywords:** knowledge, attitude, hesitancy, COVID-19, vaccine, Ethiopia

## Abstract

The current healthcare system’s efforts to reduce the spread of COVID-19 in Ethiopia and limit its effects on human lives are being hampered by hesitancy toward the COVID-19 vaccine. The aim of this study was to assess the knowledge levels, attitudes, and prevention practices of COVID-19, in the context of the level of vaccine hesitancy with other associated factors in Ethiopia. A community-based cross-sectional design with mixed-method data sources was employed. It comprised 1361 study participants for the quantitative survey, with randomly selected study participants from the studied community. This was triangulated by a purposively selected sample of 47 key informant interviews and 12 focus group discussions. The study showed that 53.9%, 55.3%, and 44.5% of participants had comprehensive knowledge, attitudes, and practices regarding COVID-19 prevention and control, respectively. Similarly, 53.9% and 47.1% of study participants had adequate knowledge and favorable attitudes toward the COVID-19 vaccine. Only 29.0% of the total survey participants had been vaccinated with at least one dose of vaccine. Of the total study participants, 64.4% were hesitant about receiving the COVID-19 vaccination. The most frequently reported reasons were a lack of trust in the vaccine (21%), doubts regarding the long-term side effects (18.1%), and refusal on religious grounds (13.6%). After adjusting for other confounding factors, geographical living arrangements, the practices of COVID-19 prevention methods, attitudes about the vaccine, vaccination status, perceived community benefit, perceived barriers toward vaccination, and self-efficacy about receiving the vaccine were significantly associated with vaccine hesitancy. Therefore, to improve vaccine coverage and reduce this high level of hesitancy, there should be specifically designed, culturally tailored health education materials and a high level of engagement from politicians, religious leaders, and other community members.

## 1. Introduction

The COVID-19 pandemic is a worldwide public health, social, and economic threat, against which efforts have been made to prevent and control its spread around the globe. This global disease infected more than 673 million population; of them, 6.7 million have already died up until January 2023. Globally, there are many therapeutic and non-therapeutic mitigation measures that have been implemented to reduce the transmission and the effect of COVID-19 on various systems [1,2]. 

COVID-19 has caused a global public health crisis affecting most countries, including Ethiopia, in various ways, including a devastating effect on the world economy and healthcare systems [1]. The COVID-19 pandemic is at a paradoxical stage, with vaccine roll-out initiated but with a still significantly elevated level of infections and deaths, while there are still delays in the acceptance or refusal of vaccines, which results in vaccine hesitancy [3,4]. Vaccine hesitancy is influenced by factors such as complacency, convenience, and confidence [4,5]. 

Vaccine hesitancy refers to a delay in the acceptance or refusal of vaccines, despite the availability of vaccination services. Vaccine hesitancy is complex and context-specific, varying across time, place, and the availability of vaccines. Vaccine attitudes can be seen as part of a continuum, ranging from total acceptance to complete refusal. Vaccine-hesitant individuals are a heterogeneous group in the middle of this continuum. Vaccine-hesitant individuals may refuse some vaccines but agree to others; they may delay vaccinations or accept vaccines but are uncertain at times [4,5]. 

According to a study conducted by Machingaidze and Wiysonge, “vaccine hesitancy is pervasive, misinformed, contagious, and is not limited to COVID-19 vaccination” [6]. Evidence showed that there is a conspiracy belief within the public consciousness that “COVID-19 vaccines are intended to inject microchips into recipients and that the vaccines are related to infertility”. Some recent studies have also reported the magnitude of vaccine hesitancy varying from 76.4% to 3.0%, indicating variabilities across different countries [7,8]. This variability could be partly due to varying perceptions and attitudes regarding the efficacy, quality, and safety of COVID-19 vaccines. Vaccine hesitancy could also be affected by sociodemographic, psychological, and cultural factors in the population. 

According to studies conducted in various countries around the world, age, parity, occupational status, gender, marital status, educational status, income, perceived risk of COVID-19 infection, being a healthcare worker, attitudes, knowledge about COVID-19, being sick with COVID-19, and the presence of chronic disease are the most important predictors of intention to receive the COVID-19 vaccine [4,5,7,9,10]. Furthermore, many myths and conspiracy theories about vaccines and COVID-19 could impair COVID-19 vaccine acceptability in society [1,11]. 

The intention to be vaccinated is also influenced by the perceived benefits of vaccination, the risks associated with COVID-19, and barriers to being vaccinated. Perceived barriers are related to lower intentions to be vaccinated against COVID-19. In different studies, age, profession, knowledge about the COVID-19 vaccine, attitudes toward the COVID-19 vaccine, and all health belief model constructs are assessed as factors of the intention to receive the COVID-19 vaccine for healthcare providers in previous studies. Educational level, working experience, and marital status were considered but were not significantly associated with the intent to receive the COVID-19 vaccine [7,10,11]. 

Despite the late start date of the pandemic and limited testing capacity, infection rates in Ethiopia have been increasing exponentially, with over 498,000 confirmed cases and more than 7572 deaths as of 23 December 2022 [12]. Evidence shows that vaccine hesitancy is due to a dearth of knowledge about the fact that vaccines are the most effective public health intervention, and have significantly reduced the burden, morbidity, and mortality of communicable diseases [13]. There are also historical, structural, and other systemic dynamics that underpin vaccine hesitancy among people who recognize the public health importance of immunization [13]. Misguided and false rumors about vaccine side effects are often spread via social media and by religious leaders [10,14,15]. Additionally, conflicts, negative experiences with the healthcare system during various epidemics, and limited trust in the government have established the perfect backdrop for vaccine-hesitant attitudes throughout the country of Ethiopia [10,16]. Moreover, the limited access to and roll-out of COVID-19 vaccines further fuel pre-existing distrust and suspicion [14]. 

In this regard, Ethiopia intends to conduct consecutive and successive national vaccination campaigns, with a focus on the urban setting, where there is a large population that may be particularly vulnerable to COVID-19 transmission due to their overcrowded living conditions. Additionally, the Ministry of Health plans to develop multidimensional communication activities and organize effective vaccination campaigns to reduce the potential fears of vaccine safety and its lack of effectiveness against COVID-19 transmission. Therefore, understanding the community’s knowledge and the extent of hesitancy is important before continuing with this multimillion-dollar investment. However, there is limited evidence in Ethiopia about the current state of COVID-19 vaccination rates and hesitancy around the country, with a particular focus on urban settings. 

The purpose of this study was to assess the knowledge levels, attitudes, and prevention practices regarding the COVID-19 vaccine and the level of vaccine hesitancy with other associated factors in selected cities in Ethiopia, including pastoralist communities. 

## 2. Materials and Methods

### 2.1. Design, Population, Study Setting, and Study Period 

A mixed-methods approach using a cross-sectional survey, in conjunction with a qualitative approach and document reviews, was used to address the objectives of the assessment. This study was conducted in Addis Ababa city, the Amhara region (Bahir Dar, Debertabor, Debreberehan, Kombolcha, Dessie, and Woledia), the Oromia region (Adama, Bishoftu, Zeway, and Shasemene), and the Sidama region (Hawassa and Yirgalem) from the agrarian part of Ethiopia. In addition, the survey included the Afar region (Semera Logia) and Somali Regional State (Jigjiga and Degahabour) from developing regional states that are pastoralist in nature. The governmental partners for this intervention include the National Ministry of Health, Regional Health Bureaus, and cities/towns’ health offices. The study was conducted between April 2022 to May 2022. The Ministry of Health of Ethiopia also planned to conduct the third-round national COVID-19 vaccination campaign in June 2022 and in the following months administered 25 million doses of different antigens all over the country. 

### 2.2. Sample Size Determination

#### 2.2.1. Quantitative Sample Size Determination

The sample size of the study was determined using a single population proportion formula, considering the objectives of the research. Since there was no previous study conducted in similar study sites, we used a 50% proportion for knowledge, attitude, and hesitancy regarding vaccines and the preventive and control methods practiced to gain the maximum sample size. In addition, the sample size calculation considered 95% CI, a 5% margin of error, a 20% non-response rate, and three design effects for its multistage sampling procedure. Finally, the calculated sample size was 1382 vaccinated and unvaccinated community members. The calculated sample size was distributed based on the age and sex of the respondent (see Table 1). 

A multistage sampling procedure was applied to recruit study participants within the community. In stage 1, 16 cities were selected randomly, and the sample was allocated based on the population proportion. In stage 2, in each city, a sample of health centers and their population catchment areas were identified. In stage 3, in each health center catchment area, one village was selected using a simple random sampling approach. In the villages, every other household was selected using a systematic random sampling procedure, and either the head of the household or the wife was considered for the interviews.

The data collection method was developed after reviewing the literature from various countries and adapting it to the context. Additionally, the study was piloted with 50 samples in the local language. Following the pilot test, the measurement tools were modified before being used for actual data collection. Data were collected electronically using tablet/smartphone applications, ODK/KOBO, where structured questionnaires with pre-coded answers were uploaded. The questionnaire was developed after reviewing the different studies in the relevant literature and was then modified, based on the project outcome indicators. The data measurement tool was developed after rigorous review for various measurements [17,18,19]. In addition, we used the health belief model to assess COVID-19 vaccine hesitancy; a systematic review indicated that health belief models (HBM) is a useful model in predicting COVID-19 vaccine hesitancy [20]. Therefore, the errors associated with this part of the tool are minimal. Additionally, the research questionnaire was pretested and modified before the actual data collection took place. The questionnaire (Table S1) included supplementary information. 

To ensure reliability and validity, our team gave intensive training to the research data collectors, including a pretest; the purpose of the research was clearly explained to study participants and the data collection was supervised by the research team. In addition, data sources and methodological triangulations were used to make the evidence reliable and valid. The local language was used during the data collection interviews to ensure the intelligibility of concepts about vaccine hesitancy; data collectors needed to speak the local language and understand the culture of the community. The research was carried out in urban areas where the public is more aware of COVID-19 and its vaccine. Furthermore, the pandemic engaged the population when it occurred, encouraging everyone to learn about its control, prevention, and vaccines. Throughout the pandemic, the government and non-governmental organizations, all media (including social media), and various sectors provided information about COVID-19; therefore, the community had no difficulty understanding the study questions. In addition, the content validity of the instrument was assessed by different experts from each study area. All the above efforts improved the study’s reliability, intelligibility, and face validity.

#### 2.2.2. Qualitative Sample Size

A purposive sample that included a total of 35 key informant interviews was conducted (with key players from an immunization program, the Ministry of Health, six Regional Health Bureaus for immunization, and eight cities/sub-cities). These key informants included eight health extension, eight health development, and nine healthcare workers who participated in the COVID-19 vaccine campaign services. In addition, 12 focus group discussion (FGDs) among the young males and females and adult men and women were included. Additionally, a semi-structured interview guide was used to facilitate the key informant interviews to obtain insightful qualitative information regarding the key issues to be examined in this research. All interviews were tape-recorded after gaining consent from each baseline survey participant.

### 2.3. Variable Measurements and Categorization

Knowledge of COVID-19’s signs and symptoms: To measure the respondents’ knowledge of the COVID-19 signs and symptoms, a total of 8 questions were posed. “Yes” responses were coded “1” and “no” responses were coded “0”. The mean score was used to categorize the study participants as knowledgeable or not knowledgeable. Those who scored above the mean score were knowledgeable, while those who scored below the mean score were considered not knowledgeable.

Knowledge of sources of COVID-19 infection: To measure the respondents’ knowledge of the sources of infection, a total of 3 questions were used. “Yes” responses were coded “1” and “no” responses were coded “0”. The mean score was used to categorize participants as knowledgeable or not knowledgeable. Those who scored above the mean score were knowledgeable, while those who scored below the mean score were considered not knowledgeable. 

Knowledge of COVID-19 infection risk groups: To measure the respondents’ knowledge of the population groups at risk, a total of 3 questions were used. “Yes” responses were coded “1” and “no” responses were coded “0”. The mean score was used to categorize participants as knowledgeable or not knowledgeable. Those who scored above the mean score were knowledgeable, while those who scored below the mean score were considered not knowledgeable.

Knowledge of COVID-19 prevention methods: To measure the respondents’ knowledge of the means of transmission, a total of 6 questions were used. “Yes” responses were coded “1” and “no” responses were coded “0”. The mean score was used to categorize participants as knowledgeable or not knowledgeable. Those who scored above the mean score were knowledgeable, while those who scored below the mean score were considered not knowledgeable. 

Overall comprehension (knowledge) of COVID-19 prevention and control options: To measure the respondents’ overall knowledge of COVID-19 prevention and control, a total of 30 questions were used. “Yes” responses were coded “1” and “no” responses were coded “0”. The mean score was used to categorize participants as knowledgeable or not knowledgeable. Those who scored above the mean score were knowledgeable, while those who scored below the mean score were considered not knowledgeable. 

Attitude toward COVID-19 prevention and control: The respondents were asked about their attitude toward COVID-19 prevention and control activities, using 7 questions with three Likert scale scales (agree, neutral, and disagree). The responses’ mean value was calculated, and those who scored above the mean were recorded as having a favorable attitude, while those who scored below the mean were recorded as not having a favorable attitude toward prevention and control activities.

COVID-19 prevention and control measures: The respondents were asked about their levels of practicing prevention and control measures, using 7 questions with 3 Likert scale scales (never, sometimes, and often). The responses’ mean value was calculated, and those who scored above the mean were recorded as having practiced the measures, while those who scored below the mean were recorded as not practicing them.

Knowledge of the COVID-19 vaccine: The respondents were asked to list all the COVID-19 prevention and control methods, and if the respondent mentioned the “COVID-19 vaccine” as a method, the respondent was considered knowledgeable. Otherwise, those who did not mention one mechanism for COVID-19 prevention and control were considered not knowledgeable. 

Attitude about the COVID-19 vaccine: The respondents were asked about their attitude toward taking the COVID-19 vaccine; “yes” responses were recorded as having a favorable attitude, while “no” responses were recorded as having an unfavorable attitude.

Vaccination status: Taking either of the available COVID-19 vaccines regardless of the stage was considered “vaccinated”.

Vaccine hesitancy: Vaccine hesitancy was measured differently for vaccinated and non-vaccinated individuals separately. Individuals who have been vaccinated are considered to have “vaccine hesitancy” if they are hesitant or regret taking the vaccine after being vaccinated, whereas, for non-vaccinated individuals, the respondent was asked about their plan to take the vaccine and if they responded, “I have no plan”, the response was recorded as “COVID-19 hesitancy”.

Perceived COVID-19 infection susceptibility: A total of 4 questions with a 5-point Likert scale (1: strongly disagree to 5: strongly agree) were used to measure perceived COVID-19 infection susceptibility. Those who scored above the mean value were categorized as having “perceived COVID-19 infection susceptibility”. Those who scored below the mean value were considered to not have perceived COVID-19 infection susceptibility. 

Perceived COVID-19 infection severity: A total of 3 questions with a 5-point Likert scale (1: strongly disagree to 5: strongly agree) were used to measure perceived COVID-19 infection severity. Those who scored higher than the mean value were classified as having a high perception of COVID-19 infection severity. Those who scored at the mean or lower were considered to have a low perception of COVID-19 infection severity.

Perceived benefits of the COVID-19 vaccine to the community: A total of 2 questions with a 5-point Likert questionnaire (1: strongly disagree to 5: strongly agree) were used to measure the perceived benefits of the COVID-19 vaccine to the community. Those who scored higher than the mean value recognized the community benefit of the COVID-19 vaccine, while those who scored lower saw no COVID-19 vaccine community benefit.

Perceived COVID-19 vaccine benefits for individuals: To assess the perceived benefits of the COVID-19 vaccine’s benefits for individuals, three questions on a five-point Likert scale (1: strongly disagree to 5: strongly agree) were used. Those who scored higher than the mean value were classified as having perceived benefits for individuals from the COVID-19 vaccine. Those who scored lower than the mean value were deemed to have no perceived COVID-19 vaccine benefit to individuals.

Perceived COVID-19 vaccine intake barriers: A total of 3 questions on a 5-point Likert scale questionnaire (1: strongly disagree to 5: strongly agree) were used to measure perceived barriers to receiving the COVID-19 vaccine. Those who scored above the mean value were categorized as having “perceived barriers” to receiving the COVID-19 vaccine. Those who scored at the mean or lower were deemed to have no perceived barrier to receiving the COVID-19 vaccine.

COVID-19 self-efficacy and the need to receive the COVID-19 vaccine: A total of 3 questions with a five-point scale Likert questionnaire (1: strongly disagree to 5: strongly agree) were used to measure the self-efficacy of the individuals regarding taking the COVID-19 vaccine. Those who scored above the mean value were categorized as having self-efficacy to receive the COVID-19 vaccine. Those who scored lower than the mean value were thought to have insufficient self-efficacy regarding receiving the COVID-19 vaccine.

### 2.4. Data Management and Analysis

#### 2.4.1. Quantitative Analysis

The quantitative data were cleaned, coded, and analyzed using STATA 16. The descriptive statistics presented tables and figures. Additionally, binary logistic regression analysis was used. The results of the multivariable binary logistic regression analyses were presented using an adjusted odds ratios, with 95% CI and a *p*-value of less than 0.05 as the level of significance. 

#### 2.4.2. Qualitative Analysis

The qualitative data analysis involved the thematic coding of transcribed and translated in-depth interviews and focus group discussions. A hybrid coding approach, which included the process of creating pre-set and emergent codes were used. Before the data collection and the coding process, pre-set codes were derived from the objectives and questions. Accordingly, a codebook was prepared which included the list of pre-codes. Additionally, the ideas, concepts, actions, relationships, and meanings that emerged from the data that were different from the pre-set codes were used as the emergent codes. The data were then analyzed using a thematic approach by conducting an ongoing content analysis. 

### 2.5. Ethical Considerations

Ethical clearance was obtained from the FMOH. Permission was secured from the respective regional, zonal, district, kebele, and community level leaders. Informed consent was obtained from each study participant after an explanation of the purpose of the assessment. Confidentiality was ensured from all data collectors and the principal investigator via coding numbers, not names, and by keeping the responses locked. 

## 3. Results

### 3.1. Sociodemographic Characteristics 

Of the total 1382 respondents calculated for the sample size, 1361 (98.5% response rate) study participants were included in the analysis. Regionally speaking, a relatively large proportion of the sample was obtained from the Somali Region 303 (22.3%) (see Table 2). Of the total number, 683 (50.2%) were male and 678 (49.8) were female respondents. More than three-quarters (76.7%) were adult men and women whose age was over 24 years, and about one-quarter (23.3%) of survey participants were youth/adolescent females and males (18–24 years old). In terms of the participants’ religious affiliation, almost half, 641 (47.1%), of the study participants were Orthodox Christian religion followers, while almost two-fifths, 536 (39.4%), were Muslim. 

In this study, 508 (37.3%) had a monthly income of less than the median ETB 6500, and 705 (51.8%) had less than the median ETB 5000 monthly expenditure. 

### 3.2. Knowledge, Attitude, and Practice Regarding COVID-19 Infection Prevention and Control

Table 3 indicates that 657 (53.9%) survey participants had adequate knowledge of COVID-19 prevention and control activities, with statistically significant differences by region and age (*p* < 0.001). The maximum percentage with adequate knowledge was observed in the Sidama Regional State (71.55%), and the minimum percentage was observed in the Somalia Regional State (37%). 

**Figure 1:**
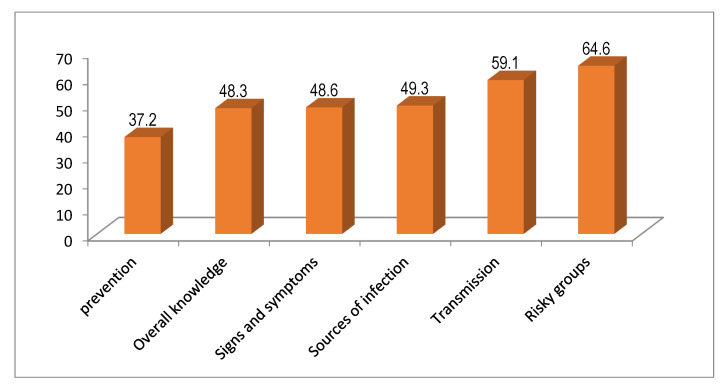
Subcomponents and prevention methods, overall knowledge level, about COVID-19 signs and symptoms, sources of infection, means of transmission, and risky groups at the time of the COVID-19 hesitancy survey, 2022.

Further analysis showed that 59% of the respondents had adequate knowledge of means of transmission and 37.2% had adequate knowledge of prevention methods against COVID-19. Figure 1 below summarizes the level of knowledge for different components regarding COVID-19 prevention and control. 

The study showed that almost three-quarters of the survey participants (74.1%) who were infected with COVID-19 may not have shown signs and symptoms. The most frequently mentioned signs and symptoms included coughing, sneezing, congestion, and a sore throat (84%). A total of six questions were used to assess the respondents’ knowledge about the means of transmission. Taking the mean value as a cutoff point to classify the respondents’ knowledge about COVID-19, about 63.6% had adequate knowledge of the transmission methods of COVID-19 infections. Regarding knowledge about COVID-19 transmission, 72.8% said that it was transmitted by touching contaminated objects, and 62.1% mentioned droplets as a means of transmission of COVID-19 infections. According to the study participants, the two most frequently mentioned conducive environments for COVID-19 were crowded places (90.5%) and people traveling across cities (89.4%). Moreover, the study respondents reported people aged 65 years or older (81.5%), with non-communicable diseases/comorbidities (59.3%), and people with known respiratory diseases (47.5%) to be at-risk population groups for COVID-19 infection severity and the related complications. About 8.5% of the study participants did not know who was at risk from COVID-19 infection. Adequate knowledge of COVID-19 prevention and control methods is essential to stop the spread of the pandemic. This study showed that most participants (88.2%) knew the importance of wearing masks, while 84.9% knew that regular hand-washing with soap and water and physical distancing (avoiding crowded/public places) were prevention control methods for reducing the transmission of COVID-19.

### 3.3. Attitudes about Infection Prevention and the Control of COVID-19 

The community’s knowledge in itself was inadequate to prevent and control COVID-19. In addition, a favorable attitude toward the COVID-19 prevention and control options was essential to ensure practice. This study showed that 753 (55.3%) of survey participants had a favorable attitude regarding COVID-19 prevention and control activities. The qualitative findings were in line with the quantitative results. Even though many people knew about vaccinations for COVID-19, they may not have been vaccinated for various reasons, such as misconceptions, issues related to politics, and high living costs in the community:

*“… there are our neighbors who think that COVID-19 does not exist and give everything to their Lord. When you inform them to take preventive measures, they consider you are working against their religion. But we have seen many people die due to COVID-19”* (21-year-old male youth from Bahirdar).

Table 4 shows the details of the different attitude domains about COVID-19 prevention methods; for instance, nearly three-quarters (74%) of respondents believed that COVID-19 was government or media propaganda, and more than half (57.5%) believed that COVID-19 was a curse.

As indicated in Table 5, below, the highest favorable attitude toward COVID-19 prevention options was observed in the Amhara Regional State and the lowest was in the Somali Regional State. About 53.4% of males and 57.2% of females had a favorable attitude toward COVID-19 prevention and control activities. Nearly half of the younger people (49.2%) and 57.2% of the adults had a favorable attitude toward COVID-19 prevention and control activities. 

#### Practices about COVID-19 Infection Prevention

Table 6 shows a total of seven questions with three Likert scale items that were used to measure the level of COVID-19 prevention practices; only 39.0% of study participants were wearing masks often, 28.7% were socially distancing often, and 39.5% were using hand sanitizer often to prevent the spread of COVID-19. 

This study showed that 55.6% of the respondents followed inadequate COVID-19 prevention practices. The highest levels of COVID-19 prevention practices were observed in the Amhara Regional State (73.08), and the lowest were observed in the Somali Regional State (32.3%) (*p* < 0.001). 

### 3.4. Level of COVID-19 Testing

Of the total study participants, 33.8% were tested for COVID-19 after the pandemic period began; of those who were tested, 36 (2.6%) were positive for COVID-19. The remainder (66.2%) were not tested for COVID-19 for different reasons. More than half (55.3%) mentioned an absence of COVID-19 symptoms, fearing the test (27.5%), and a lack of trust (15.5%), which were the main reasons for not taking COVID-19 tests.

### 3.5. Knowledge, Attitude, and Vaccination Practices Regarding the COVID-19 Vaccine 

Community knowledge and attitudes about the COVID-19 vaccine are a prerequisite to taking the vaccine. In this study, 733 (53.9%) had adequate knowledge about the COVID-19 vaccine as one of the infection prevention methods, and 720 (52.9%) had a favorable attitude about the importance of the COVID-19 vaccine as a COVID-19 prevention and control strategy. A statistically significant association was observed among the regions (see Table 7) and from having adequate knowledge about the COVID-19 vaccine as a prevention option (*p* < 0.001). Among the Regional States, the highest knowledge level was observed in the Sidama Regional State (75.65%), and the lowest frequency level was observed in the Somali Regional State (33.0%).

Forty-seven percent of respondents had a favorable attitude toward the COVID-19 vaccine. Regional variations regarding attitudes toward the COVID-19 vaccine were observed; the highest favorable attitude about the vaccine was observed in the Amhara Regional State (52.56%), while the lowest was observed in the Oromia region (39.01%). 

Only 29.0% of the total survey participants were vaccinated with at least one dose of vaccine since the vaccine had been introduced. Regional variations were observed in vaccination status; the highest vaccination status level was observed in the Sidam Regional State (55%) and the lowest in the Somali Regional State (14.5%). The vaccination status was also higher among adult population groups (32.0%) in comparison to young people (20.0%). As indicated in Table 7 below, there is a statistical difference in vaccination status based on the region (*p* < 0.001) and age (*p* < 0.0.001) of the survey participants. 

The proportion of unvaccinated study respondents was as high as 71%, for different reasons. According to the self-reported reasons, these included a lack of trust in the vaccine (18.0%), religious reasons (16.8%), lack of time to attend the appointment (11.7%), and lack of access or eligibility issues with the vaccine (38%). Of those vaccinated study participants, 65.1% of them did not know what type of vaccine they received. Only 15.7%, 13.2%, 5.3%, and 0.8% of survey participants knew that they received the Johnson and Johnson, Astra Zeneca, and Pfizer vaccines, respectively. Only 8.4% of the study participants chose the type of vaccine and the remaining received whatever vaccine was available without knowing the existing alternatives. 

### 3.6. Self-Reported Vaccine Adverse Effects 

**Figure 2:**
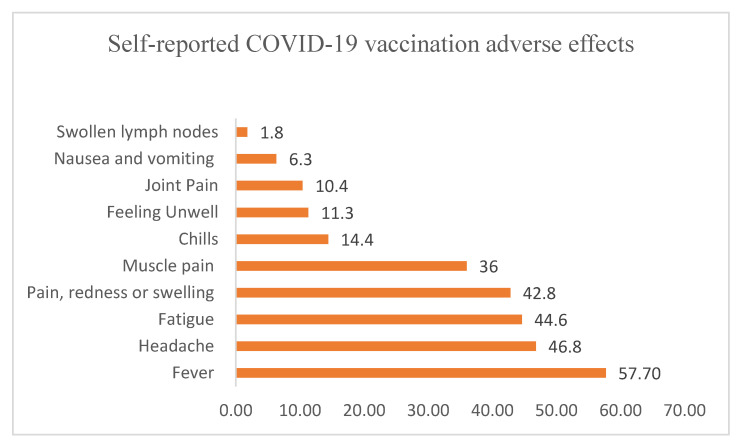
Self-reported COVID-19 vaccination adverse effects among vaccinated individuals at the time of the COVID-19 vaccine hesitancy survey, Ethiopia, 2022.

Of the total vaccinated survey participants, 222 (56.2%) reported vaccine-related mild to moderate adverse effects in this survey. As indicated in Figure 2, of those who reported vaccine adverse effects, 57.7%, 46.8%, and 44.6% reported fever, headache, and fatigue as adverse effects, respectively. 

### 3.7. COVID-19 Vaccine Hesitancy 

**Figure 3:**
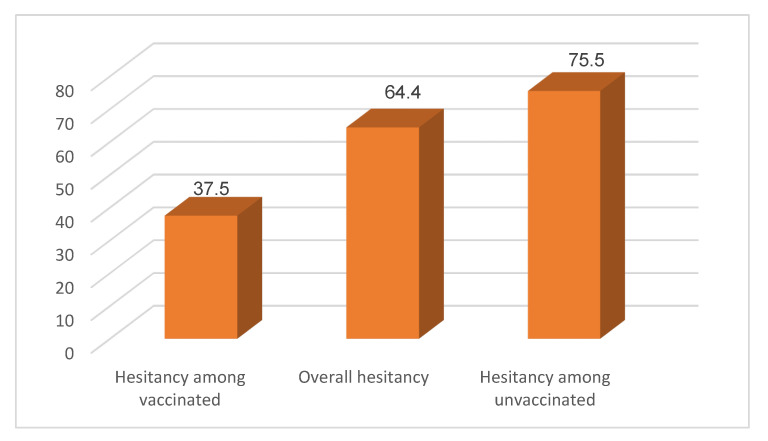
Level of vaccine hesitancy among survey participants at the time of the COVID-19 vaccine hesitancy survey, Ethiopia, 2022.

The findings of this study identify that of the 966 unvaccinated individuals, 729 (75.5%) hesitated to receive the vaccine. Similarly, the level of regret after receiving the COVID-19 vaccination was assessed for the vaccinated individuals. Of those vaccinated individuals, 148 (37.5%) regretted receiving the vaccine (Figure 3). 

We disaggregated vaccine hesitancy prevalence by the region, gender, and age of the survey participants. The highest frequency of vaccine hesitancy was observed in the Afar region (78.5%), while the lowest frequency was observed in the Sidama region (47.82%). As indicated in Table 8 below, there was a statistical difference in vaccine hesitancy based on region, but there was no statistically significant difference based on the age and gender of survey participants in the study area. 

### 3.8. Reasons for Vaccine Hesitancy 

**Figure 4:**
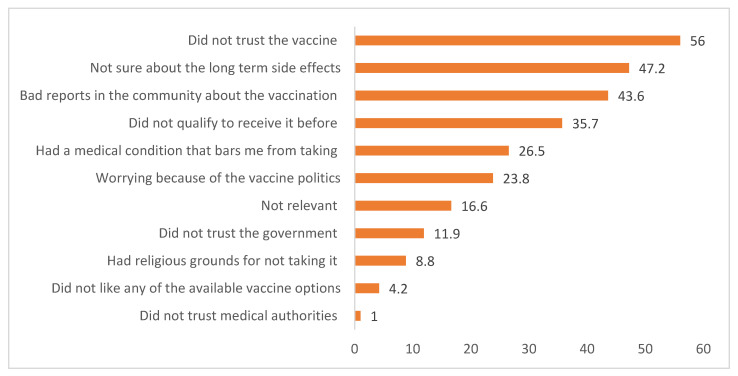
Reasons for vaccine hesitancy among unvaccinated individuals during COVID-19 hesitancy survey, Ethiopia, 2022.

Of those vaccinated individuals, 14.4% were not interested in recommending the vaccine to other people. Vaccinated survey participants were asked about their perceptions of the main reasons of those in the community who did not receive the vaccine. It was reported that 40.2% did not know about the vaccine, while the remaining 39.2% had no trust in the vaccine. Similarly, unvaccinated study participants (966) gave different reasons for being hesitant, including not trusting the vaccine (56%), the fear of long-term effects (47.2%), and negative reports about the vaccine (43.6%). The details are indicated in Figure 4 below. 

Of the total unvaccinated survey participants, 138 (25.1%) planned to receive the vaccine in the near future. Among those who showed an interest in receiving the vaccine, 75.4% did not know which vaccine they should receive, and about 11.6% were interested in receiving the Johnson & Johnson COVID-19 vaccines. 

### 3.9. Myths and Misconceptions about the COVID-19 Vaccine

Political conspiracy: In addition to being suspicious of the vaccine’s efficacy, the community had heard various rumors and misconceptions, such as politicians’ agendas and religious views, resulting in the low utilization of the vaccine. The above reasons make vaccination programs challenging. For example, a 30-year-old female EPI coordinator and KII participant from FMOH stated:

*“Different rumors are circulating in the community. For example, the community raised issues like the vaccine is fake, the vaccine is political, the politicians have brought it for themselves, COVID-19 does not exist in Ethiopia, the vaccine is 666, and the vaccine is micro-chips. Things went wrong, and we had a big challenge when we started the vaccination programs”* (EPI coordinator KII participant from FMOH).

Religion: According to the qualitative findings, there was a tendency to give different reasons for linking the COVID-19 vaccine to religion and belief. One of the KII participants from a health center in Yeka sub-city stated: “There is a strong belief in the community that what saves them is not wearing face masks or taking the vaccine, but it is their belief/religion”.

The above statement also narrated the issue of religion and the COVID-19 vaccine by a community FGD participant; a 27-year-old male FGD participant from Batu stated:

*“….as to my understanding, it is also related to religious reasons. I believe in God, and I live by the will of God rather than the vaccine. So, why do I take the vaccine?”*

Fear of Side Effects: The qualitative findings indicated that the fear of side effects was one of the main reasons for not taking the vaccine. Fear of vaccination is mentioned as one of the main reasons for being hesitant to receive COVID-19 vaccinations at all levels. An adult 45-year-old FGD participant from Bahir Dar stated “I am afraid of being vaccinated. I did not get vaccinated because I have seen many people get sick after taking the vaccine”.

### 3.10. Perceptions about COVID-19 Vaccine Hesitancy

#### 3.10.1. Perceived Susceptibility to COVID-19 Infection 

Those who perceived their susceptibility to the COVID-19 infection were more likely to receive the vaccine. Of the total survey participants, 754 (55.4%) had a perceived susceptibility to COVID-19 infection. Regional variations were observed; the maximum perceived susceptibility was observed in the Oromia region (81%) and the minimum susceptibility was observed in the Afar Regional State (12.4%) (*p* < 0.001). 

#### 3.10.2. Perceived Severity of COVID-19 Infection 

In the survey, 44.5% considered that COVID-19 infection and its complications were not severe. The qualitative study indicated a low risk perception in the community regarding COVID-19, affecting the vaccine intake. “Another main challenge is a low-risk perception on COVID-19 infection in our society. People believe that I already had a COVID-19 infection even though it did not show signs and symptoms, and I have no risk of reinfection again. So, why do I take [the] COVID-19 vaccine?” (KII at MOH). 

Similarly, participants mentioned that there was no problem concerning COVID-19 prevention and control measures. The problem is associated with the low perceived risk of the disease. “I don’t think there is a significant knowledge gap about how COVID-19 is transmitted and the prevention measures. People hear about it from many media. But we can see a huge gap regarding the preventive measures. There is a low risk perception among the community. As a result of this, hand washing, hand sanitizer usage, and wearing of face mask are very poor” (KII EPI unit at Hawassa).

Another 19-year-old respondent from Addis Ababa added, “Many people see the disease as simple. The previous panic and anxiety related to the disease have been lost. This has a great impact on the uptake of COVID-19 vaccines”. 

### 3.11. Perceived Individual and Community Benefits Regarding COVID-19 Vaccines

Evidence has shown that when individuals believe that receiving the vaccine is important to prevent COVID-19 from spreading in their community, the probability of being vaccinated is very high. As indicated in Table 9 and Table 10 of the total participants, 794 (54.2%) perceived that receiving the COVID-19 vaccine had individual benefits, and 739 perceived that receiving COVID-19 had community benefits. Regional variations were observed in the perception of the study participants regarding the importance of the vaccine to the community. The highest (64.4%) importance was observed in Addis Ababa, and the lowest (44%) was observed in the Sidama Regional State (*p* < 0.001). Similarly, regional variations were observed in the perceptions of the respondents about the importance of the vaccine on the individual; the highest (70.4%) was observed in Addis Ababa, and the lowest (42.4%) in the Afar Regional State (*p* < 0.000) (Table 9). 

### 3.12. Perceived Barriers to Receiving COVID-19 Vaccines

This study showed that perceived barriers to receiving COVID-19 vaccines were reported by 836 (61.4%) respondents. Regional variations were observed in perceived barriers to COVID-19 vaccine uptake (*p* < 0.001); the highest (75.9%) perceived barrier to receiving the vaccine was observed in the Afar Region and the lowest (42.7%) in the Amhara Region (*p* < 0.001). 

### 3.13. Self-Efficacy about the COVID-19 Vaccine

The assessment measured self-efficacy about receiving COVID-19 vaccines, and 720 (52.9%) had high self-efficacy about receiving the vaccine. The highest self-efficacy was observed in Addis Ababa (56.9%) and Sidama (56.9%) Regional States, and the lowest was observed in the Amhara Regional States (42.7%). A statistically significant association was observed between the regions and self-efficacy about the vaccine (*p* < 0.001). 

### 3.14. Factors Associated with Vaccine Hesitancy 

Using the city/sub-city administration as a cluster, data analysis was tested for a multilevel binary logistic regression model to identify the factors associated with vaccine hesitancy. However, in the empty model, the intra-class correlation coefficient (ICC) (1.07%) was less than 10%; in this case, classical regression was recommended to identify the factors associated with COVID-19 vaccine hesitancy. 

To control the confounder variables and identify the independent factors with the dependent variable of vaccine hesitancy, variables with a *p*-value of less than 0.05 during the bivariate binary logistic regression were included in the model. The variables included in the final model were the respondent’s region, educational status, prevention practices, attitudes toward the COVID-19 vaccine, vaccination status, perceived susceptibility, severity, individual benefit, perceived barriers, and self-efficacy. 

Using a *p*-value of less than 0.05, region, practices about prevention methods, attitudes about the vaccine, vaccination status, perceived community benefit, perceived barriers regarding vaccination, and self-efficacy were significantly associated with vaccine hesitancy. Respondents from the Sidama Regional state had odds of 53% (AOR, 95% CI: 0.47 (0.26, 0.84) of being less likely to be hesitant than residents from the Somali Regional state. The odds of study participants being from the Afar Regional state were 1.96 (AOR, 95% CI: 1.96 (1.19, 3.23)) times more likely to be hesitant in comparison with study participants from the Somali Regional State. 

The odds of study participants that had inadequate preventive practices showed that they were 1.37 (AOR, 95 CI: (1.01, 1.87)) times more likely to be hesitant than study participants with adequate preventive practices. In addition, the odds of being hesitant among study participants with an unfavorable attitude toward the vaccine showed that they were 1.76 (AOR, 95%CI: 1.32, 2.37) times more likely to be hesitant to receive the COVID-19 vaccine than study participants with a favorable attitude. The odds of non-vaccinated individuals being hesitant showed that they were 1.72 (AOR, 95%CI: 1.27, 2.34) times more likely to be hesitant than vaccinated individuals. The odds of study participants having lower self-efficacy were about 8.79 (AOR, 95% CI: 5.48, 14.07) times more likely to be hesitant than those with higher self-efficacy (See Table 10). 

**Table 10:** Multivariable binary logistic regression results from the COVID-19 vaccine hesitancy survey, Ethiopia, 2022.

Variable	Category	Hesitancy		COR, 95% CI	AOR, 95% CI	
		No	Yes			Effect Size (Cohen’s f ^2^)
Region	Addis Ababa	102	151	0.63 (0.44, 0.89)	0.83 (0.51, 1.35)	0.46
	Amhara	78	156	0.84 (0.59, 1.22)	0.91 (0.55,1.50)	
	Sidama	61	55	0.38 (0.25, 0.59)	0.47 (0.26, 0.84) *	
	Oromia	112	152	0.57 (0.41, 0.81)	0.83 (0.52,1.34)	
	Afar	41	150	1.55 (1.01, 2.36)	1.96 (1.19,3.23) **	
	Somali	90	213	1	1	
Educational status	No formal education	73	160	1.504 (1.08,2.10)	1.07 (0.71, 1.62)	
	Primary (1–8 grade)	53	93	1.204 (0.82, 1.77)	1.17 (0.72,1.90)	
	Secondary (9–12 grade)	172	353	1.409 (1.08, 1.83)	1.31 (0.96,1.79)	
	Certificate and above	186	271	1	1	
Practice on prevention	Not adequate practice	199	557	2.49 (1.99, 3.13)	1.37 (1.01,1.87) **	0.066
	Adequate practice	285	320	1	1	
Attitude about vaccine	Not favorable attitude	157	563	3.73 (2.95,4.72)	1.76 (1.32, 2.37) ***	0.64 ****
	Favorable attitude	327	314	1		
Vaccination status	Not vaccinated	237	729	5.13 (3.99,6.60)	1.72 (1.27,2.34) ***	0.85 ****
	Vaccinated	247	148	1		
Perceived susceptibility	Below mean score	165	442	1.96 (1.56,2.47)	0.86 (0.62, 1.21)	
	Above mean score	319	435			
Perceived severity	Below mean score	127	478	3.37 (2.64,4.29)	1.34 (1.23,1.49) ***	0.57 ****
	Above mean score	357	399	1		
Perceived individual benefits	Below mean score	24	166	4.475 (2.87,6.98)	1.25 (1.12,2.15) ***	0.56 ****
	Above mean score	460	711	1		
Perceived barriers	Below mean score	103	422	3.431 (2.66,4.43)	0.73 (0.49, 1.09)	0.56 ****
	Above mean score	381	455	1		
Self-efficacy	Below mean score	68	573	11.53 (8.61,15.44)	8.79 (5.48, 14.07) ***	1.13 ****
	Above mean score	416	304	1	1	

* *p* < 0.05, ** *p* < 0.01, *** *p* < 0.001. **** f ^2^ = large effect size (> 0.5).

## 4. Discussion

The main purpose of the current COVID-19 vaccine hesitancy survey was to assess the knowledge level, attitudes, and prevention and control practices regarding COVID-19, and the level of vaccine hesitancy and associated factors in Ethiopia. This study is one of the few community-based studies to assess the Ethiopian community’s perceptions about COVID-19 vaccine hesitancy and the associated factors using a large sample size covering urban settings from six regions in the country.

A variety of measures have been implemented to control the pandemic by improving the community knowledge, attitudes, and practices regarding COVID-19 prevention and control [16], despite the findings of this study indicating that only 53.9% of participants had adequate knowledge about COVID-19 prevention and control activities. Similarly, the qualitative findings highlight the finding that the community was not making COVID-19 a priority issue. Studies in Ethiopia inconsistently reported the magnitude of having adequate knowledge about COVID-19 prevention and control, which ranged from 25.0% to 88.2% [21]. This difference might be related to the study population characteristics, where the current study is a community-based study and the other study included preparatory and university students who have better media access to learn about COVID-19, including school-based interventions. In addition, our study included a large sample size with various population dynamics across the country, in comparison to other studies. In contrast, the current study findings about the knowledge of the community regarding COVID-19 prevention and control is lower than studies conducted in China (82.34%) [22] among undergraduate students and with study respondents from Cameroni (84.19%) [23]. This might be attributed to contextual differences, such as information access and literacy levels between countries. 

Favorable community attitudes toward COVID-19 prevention and control play a major role in designing effective community-based interventions, including COVID-19 vaccination as one prevention and control strategy. However, in this study, 55.3% of survey participants had a favorable attitude toward COVID-19 prevention and control activities. The qualitative findings support the argument that the community considered COVID-19 to be a political agenda to sell the vaccine. This implies that a significant number of individuals had negative attitudes about the prevention and control activities of COVID-19, including receiving the vaccine as a prevention and control strategy. 

Different studies reported inconsistent findings on the magnitude of favorable attitudes toward COVID-19 prevention and control activities, which ranged from 56.6% to 94.8% [21]. This variability could be explained because of population differences between healthcare workers and students. Both healthcare workers and students are exposed to COVID-19 information because of the nature of their susceptibility to infection.

In the current study, only 44.5% of the study participants used adequate COVID-19 prevention and control practices, including COVID-19 vaccination. Similar to knowledge and attitudes, various studies in Ethiopia reported inconsistent findings on the magnitude of practices about COVID-19 prevention and control activities. The current study’s findings about COVID-19 prevention and control activities are higher than studies conducted in other parts of Ethiopia, such as Arbaminch (23.5%) [21] and Gedeo (39.5) [24]. These discrepancies might be due to differences in community awareness as a result of mass media and social media.

In terms of the efforts regarding COVID-19 prevention and control battles, the high proportion of the community with adequate knowledge and favorable attitudes is a prerequisite to receiving the vaccine. However, in the current study, only 53.9% of study participants had adequate knowledge about the COVID-19 vaccine as one of the infection prevention approaches, and only 47.1% of the study participants had a favorable attitude toward the importance of the COVID-19 vaccine as a COVID-19 prevention and control strategy. These findings indicate a wide knowledge and attitude gap about COVID-19 vaccination, which may be attributed to a low perceived susceptibility among pastoralist study groups. This implies that efforts should be targeted at increasing knowledge levels and building positive attitudes in parallel with vaccination campaigns in the community. These findings suggest that intensive advocacy, social mobilization, and community awareness activities in collaboration with partners should be strengthened. 

The distribution and intake of vaccines are shaped by challenging political, economic, social, diplomatic, and health-related matters; in this study, less than one-third of the total survey participants were vaccinated with at least the first Pfizer dose, the Johnson & Johnson, SINO, or AstraZeneca vaccine since the vaccine was first introduced in Ethiopia on 13 March 2021. Having a comorbidity, vaccine promotion campaigns, and the level of perceived risk for COVID-19 infections were shown as justifications for receiving the vaccine. This study’s results are slightly higher than the national level of coverage, where more than 21.5 million people have received at least one dose and more than 20.5 million people have been fully vaccinated [2]. This implies that extra effort should be made to increase vaccine uptake, including expanding vaccination sites and ensuring the effective use of available stocks. 

In the current study, which has been conducted in major towns and cities in each region, 64.4% of the study participants were hesitant about receiving the COVID-19 vaccination. A study among healthcare workers in Ethiopia indicated similar findings, which showed that 60.3% of healthcare workers were hesitant about receiving the COVID-19 vaccine [25]. Alternatively, our study showed a higher uptake of vaccines in Addis Ababa [3] in comparison to other parts of Ethiopia and abroad, where the study participants were not willing to get vaccinated. For example, areas of Arbaminch (23.5%) and Gedeo (39.5%), and other countries such as Ghana (34.9%), and Malaysia (22.7%) showed higher levels of vaccine hesitancy [3,7]. This difference might be associated with major cities, where the inhabitants have a better understanding of COVID-19 infection risks and the community has more information. Different reasons were mentioned for being hesitant, including s lack of trust regarding the vaccine (21%), doubts about the long-term side effects (18.1%), and religious grounds (13.6%). The qualitative part of the study explored the reasons for being hesitant, including misconceptions and rumors about the COVID-19 vaccine, the fear of side effects, a lack of awareness, and misinformation and misunderstandings about the vaccine. Rumors and conspiracy theories can contribute to vaccine anxiety and those rumors about vaccination campaigns being used for political purposes are not new [26]. Community conversations with the close involvement of stakeholders, including community elders, religious leaders, and the administrative structure are necessary for groups to work closely to decrease vaccine hesitancy. 

An individual’s desire to avoid illness related to COVID-19 depends on the belief that receiving the vaccine will prevent, or cure, the illness or reduce the severity of the disease. Ultimately, an individual’s course of action to be vaccinated often depends on the person’s perceptions of their susceptibility to infection, disease severity, individual and community level benefits, barriers regarding being vaccinated, and self-efficacy [4,20]. In this study, only 55.4% had perceived their susceptibility regarding the COVID-19 infection, 55.5% had perceived that the COVID-19 infection was severe, 54.2% perceived that the COVID-19 vaccine had benefits, 61.4% harbored mental barriers regarding being vaccinated, and 52.9% of the study participants had positive self-efficacy about receiving the COVID-19 vaccine. 

Different factors showed a significant association with vaccine hesitancy via the regression analysis. Those who had low levels of practice regarding COVID-19 prevention and control and poor attitudes had a high probability of being hesitant, which finding had been supported by a study conducted in Addis Ababa [3]. The geographical area where each respondent lived was also one of the factors that affected vaccine hesitancy, which may have created vaccine coverage imbalances among the regional states. Those who believed that being vaccinated had an importance to the community had a lower chance of being hesitant, which reflects the herd immunity concept of immunization. To safely achieve herd immunity against COVID-19, a substantial proportion of the population would need to be vaccinated, lowering the overall amount of virus to be available to spread to the whole population. Promoting the importance of herd immunity is helpful to keep vulnerable groups who cannot be vaccinated safely (for example, due to health conditions such as allergic reactions to the vaccine) so that they remain protected from the disease [12,27,28,29]. The presence of barriers was a significant variable that affected COVID-19 vaccine hesitancy and the qualitative study highlights this point. The findings of this study highlight a combination of factors that restrict the rapid uptake of vaccines. 

Self-efficacy was an important factor in wanting to receive the vaccine and was significantly associated with vaccine hesitancy in the current study. Similarly, a study conducted among university students showed that self-efficacy significantly predicts adherence to precautionary measures regarding COVID-19 prevention and control measures, including receiving the vaccine [27,28,29]. 

### 4.1. Implications of the Study 

This study is crucial to deepen our understanding of COVID-19 vaccine hesitancy. The study findings from this survey can inform policymakers and administrators about the opportunities and constraints in vaccinating the community. The findings may thus contribute to developing a strategy for controlling the pandemic by addressing those factors significantly affecting vaccination uptake. Thus, it helps vaccination program implementers and decision-makers to make wise investments to reduce the impact of COVID-19 and enhance vaccination uptake. This includes contextualizing messaging during intervention or other interventions. Moreover, this study provides policymakers and program implementers with important insights into the effective review of COVID-19 policies and the allocation of resources, both human and financial, to improve the COVID-19 situation, with a special emphasis on vaccination.

### 4.2. Strengths and Limitations of the Study

This study’s strengths included the use of standard measurement tools after pilot testing with a large sample size and the use of rigorous analysis at various stages. A large sample size was considered and, based on population structure, the proportional allocation was attended by age (youths and adults) and gender (male and female). Additionally, the study samples are from diverse study settings, including agrarian and pastoralist communities. The limitation of this study is that it shows no causality of effects, due to the nature of the cross-sectional design. Additionally, this study is only limited to urban settings but does not address some of the factors related to urban settings, such as refugees and internally displaced people. Hence, the generalization is limited to people residing in the urban setting of Ethiopia.

## 5. Conclusions

Based on this study, the knowledge, attitudes, and practice levels regarding COVID-19 prevention and control are inadequate for reducing hesitancy about COVID-19 vaccinations and improving vaccine coverage. There is a statistically significant association between the knowledge level, attitudes, and practices regarding COVID-19 prevention and control and the gender and age of the respondents. Variations were observed among regional states in terms of knowledge, attitudes, and practices regarding COVID-19 prevention and control. A low level of knowledge was reported from the pastoralist region. 

The vaccination status is very low in Ethiopian urban cities across the country. Regardless of the optimum distribution of vaccines in the urban setting, more than half of the survey participants were hesitant about receiving the COVID-19 vaccine, and different reasons and misconceptions were reported. Educational status, knowledge levels regarding prevention, prevention practices, unfavorable attitudes toward the vaccine, receiving the vaccine, perceived community benefits, perceived barriers regarding receiving the vaccine, and self-efficacy were identified as factors associated with vaccine hesitancy at a *p*-value of less than 0.05, after controlling for potential confounder variables. Therefore, to improve vaccine coverage and reduce the current high level of COVID-19 vaccine hesitancy, we recommend making efforts to improve the knowledge level, attitudes, and control and prevention practices regarding the importance of receiving the COVID-19 vaccine. Rumors, misunderstandings, and misconceptions were reported as a reason for vaccine hesitancy; there should be vaccine promotion activities in collaboration with local institutions, including religious institutions. Establishing participatory engagement and open debates that include minorities and marginalized communities should be a priority early in the vaccine roll-out. Furthermore, there should be clear communication protocols for communicating with the public about adverse events. Additionally, before commencing massive immunization programs, an effective communication strategy should be designed. This strategy should consider all the different languages being spoken within a country and focus on context-specific messaging to build upon personal and positive stories and promotion on social media. Moreover, this study provides policymakers and program implementers with important insights for the effective review of the COVID-19 policies, for the allocation of financial resources to improve COVID-19 awareness, with a special emphasis on vaccination. In summary, to achieve high acceptance and uptake, evidence-based and behaviorally informed strategies should be designed and used, for instance, focusing on building trust in COVID-19 vaccines before people form an opinion against them, highlighting the consequences of inaction during consultations with health professionals, and emphasizing the social benefits of vaccination. Other strategies, such as engaging the religious leaders and gatekeepers, including the health extension workers in Ethiopia, are critical. 

## Figures and Tables

**Table 1:** Number of cities in each region, number of districts, and sample size distribution, shown according to region sub-city, district, sex, and age.

Region	Regional Population	Study Urban Population	Sample Size		City/Sub City	Sample Size		Sex		Age Category	
			N	%		N	%				N
Addis Ababa	3,434,000	655,430	253	18.6	Yeka	90	6.6	Male	45	18–24 years old	18
								Female	45	Over 24 years	72
					Nifas silik lafto	74	5.4	Male	40	18–24 years old	16
								Female	34	Over 24 years	58
					Gulelie	89	6.5	Male	44	18–24 years old	24
								Female	45	Over 24 years	59
Amhara	21,134,988	2,433,723	234	17.2	Bahir Dar	143	10.5	Male	66	18–24 years old	32
								Female	77	Over 24 years	111
					Debre Birhan	90	6.6	Male	47	18–24 years old	19
								Female	43	Over 24 years	71
Sidama	3,200,000	191,171	116	8.5	Hawassa	116	8.5	Male	58	18–24 years old	23
								Female	58	Over 24 years	93
Oromia	35,467,001	243,661	264	19.4	Adama	153	11.2	Male	76	18–24 years old	23
								Female	77	Over 24 years	130
					Batu	111	8.2	Male	54	18–24 years old	23
								Female	57	Over 24 years	88
Afar	1,812,002	23,300	191	14.0	Semera Logiya	191	14.0	Male	93	18–24 years old	48
								Female	98	Over 24 years	143
Somali	5,748,998	115,817	303	22.3	Jijiga	233	17.1	Male	128	18–24 years old	73
								Female	105	Over 24 years	160
					Degahabour	70	5.2	Male	31	18–24 years old	19
								Female	39	Over 24 years	51
Total			1361	100		1361	100		1361		1361

**Table 2:** Socio-demographic characteristics of COVID-19 vaccine hesitancy survey, Ethiopia, 2022.

Variable	Category	Frequency (%)
Region	Addis Ababa	253 (18.6)
	Amhara	234 (17.2)
	Oromia	116 (8.5)
	Sidama	264 (19.4)
	Afar	191 (14.0)
	Somali	303 (22.3)
Gender	Male	683 (50.2)
	Female	678 (49.8)
Age	18–24 years	317 (23.3)
	Over 24 years	1044 (76.7)
Religious status	Orthodox	641 (47.1)
	Muslim	536 (39.4)
	Protestant	170 (12.5)
	Other *	14 (1.0)
Educational status	No formal education	233 (17.1)
	Primary school	146 (10.7)
	secondary school	525 (38.6)
	certificate and above	457 (33.6)
Occupation	Merchant	282 (20.7)
	Housewife	216 (15.9)
	Government employed	247 (18.1)
	Private	144 (10.6)
	Student	306 (22.5)
	No job	103 (7.6)
	Farmer	21 (1.5)
	Other **	42 (3.1)
Marital status	Married	610 (44.8)
	Single	605 (44.4)
	Divorced	73 (5.4)
	widowed	73 (5.4)
Partner educational status (N = 610)	No formal education	156 (25.6)
	Primary school	83 (13.6)
	secondary school	100 (16.4)
	certificate and above	141 (23.1)
Partner occupation (N = 610)	Merchant	124 (20.3)
	Housewife	172 (28.2)
	Government employee	152 (24.9)
	Privately employed	100 (16.4)
	No job	10 (1.6)
	Farmer	21 (3.4)
	Student	10 (1.6)
	Other	21 (3.4)
Family size	Fewer than 3 in the family	469 (34.5)
	3–5 in the family	532 (39.1)
	More than five in the family	360 (26.5)
Monthly Income Median = ETB 6500	Less than median	508 (37.3)
	Median and above	853 (62.7)
Monthly expenditure Median = ETB 5000	Less than median	705 (51.8)
	Median and above	656 (48.2)

* Waqifeta and Catholic, ** student, farmer, daily laborer.

**Table 3:** Level of knowledge by region, sex, and age regarding COVID-19 prevention methods at the time of the COVID-19 hesitancy survey in Ethiopia, 2022.

Variable	Category	Adequate Knowledge on Prevention		*p*-Value
		No (%)	Yes (%)	
Region	Addis Ababa	145 (57.3)	108 (42.7)	*p* < 0.001
	Amhara	110 (47.0)	124 (53)	
	Sidama	33 (28.44)	83 (71.55)	
	Oromia	148 (56.0)	116 (43.94)	
	Afar	77 (40.3)	114 (59.7)	
	Somali	191 (63.04)	112 (36.96)	
Sex	Male	361 (52.9)	322 (47.1)	*p* = 0.589
	Female	343 (50.6)	335 (48.2)	
Age	Youth	141 (44.5)	176 (55.5)	*p* < 0.01
	Adult	563 (53.9)	481 (46.1)	

**Table 4:** Attitudes for each item of the questionnaire about COVID-19 prevention methods at the time of the COVID-19 hesitancy survey, Ethiopia, 2022.

Variable	Category	Frequency (%)
Preventing COVID-19 will be more difficult if people or other communities do not keep up with the information related to prevention	Agree	728 (53.5)
	Don’t know	138 (10.1)
	disagree	495 (36.4)
Preventing COVID-19 will be more difficult if people or other communities no longer need to worry about contracting COVID-19	Agree	748 (54.8)
	Don’t know	145 (10.7)
	disagree	470 (34.5)
Preventing COVID-19 will be more difficult if people or other communities are easily influenced by negative news	Agree	731 (53.7)
	Don’t know	159 (11.7)
	disagree	471 (34.6)
I feel that persons experiencing the symptoms or persons infected should be motivated to implement COVID-19 prevention measures and ensure a healthy life.	Agree	724 (53.2)
	Don’t know	125 (9.2)
	disagree	512 (37.6)
I feel that COVID-19 is government or media propaganda.	Agree	1007 (74.0)
	Don’t know	162 (11.9)
	disagree	192 (14.1)
I feel that COVID-19 is a curse.	Agree	783 (57.5)
	Don’t know	267 (19.6)
	disagree	311 (22.9)

**Table 5:** The levels of attitudes by region, sex, and age toward COVID-19 prevention at the time of the COVID-19 hesitancy baseline survey in Ethiopia, 2022.

Variable	Category	Favorable Attitude on Prevention		*p*-Value
		No (%)	Yes (%)	
Region	Addis Ababa	45 (17.8)	208 (82.2)	*p* < 0.001
	Amhara	23 (9.8)	211 (90.2)	
	Sidama	19 (16.4)	97 (83.6)	
	Oromia	44 (16.7)	220 (83.3)	
	Afar	15 (26.8)	41 (73.2)	
	Somali	202 (66.2)	103 (33.8)	
Gender	Male	318 (46.6)	365 (53.4)	*p* < 0.001
	Female	290 (42.8)	388 (57.2)	
Age	Youth	161 (50.8)	156 (49.2)	*p* < 0.01
	Adult	447 (42.8)	597 (57.2)	

**Table 6:** COVID-19 prevention practices at the time of the COVID-19 vaccine hesitancy surveys, Ethiopia, 2022.

Variable	Category	Frequency (%)
Wear a mask in crowded or public places	Never	363 (26.7)
	Sometimes	467 (34.3)
	Often	531 (39)
Keep distance between (physical distance) in crowded or public places	Never	541 (39.8)
	Sometimes	430 (31.6)
	Often	390 (28.7)
Use hand sanitizer and take a bath after going to a crowded or public place	Never	381 (28)
	Sometimes	442 (32.5)
	Often	539 (39.5)
Change your clothes after going to a crowded or public place	Never	621 (45.6)
	Sometimes	334 (24.5)
	Often	406 (29.8)
Carry out a campaign to prevent the spread of COVID-19 by providing a direct example in daily activity	Never	528 (38.8)
	Sometimes	426 (31.3)
	Often	407 (29.9)
Eat fruits and vegetables in the last few days	Never	146 (10.7)
	Sometimes	509 (37.4)
	Often	706 (51.9)
Exercise routinely	Never	624 (45.8)
	Sometimes	340 (25)
	Often	397 (29.2)

**Table 7:** The level of COVID-19 vaccination status by region, sex, and age regarding COVID-19 prevention attitudes at the time of the COVID-19 hesitancy baseline survey in Ethiopia, 2022.

Variable	Category	Vaccination Status		*p*-Value
		No (%)	Yes (%)	
Region	Addis Ababa	163 (64.43)	90 (35.57)	*p* < 0.001
	Amhara	184 (78.63)	50 (21.37)	
	Sidama	52 (45.02)	63 (54.98)	
	Oromia	150 (58.82)	114 (43.18)	
	Afar	154 (80.6)	37 (19.4)	
	Somali	262 (86)	41 (14.5)	
Gender	Male	493 (72.2)	190 (27.8)	*p* = 0.325
	Female	473 (69.8)	205 (30.2)	
Age	Youth	256 (80)	61 (20.0)	*p* < 0.001
	Adult	710 (68)	334 (32)	

**Table 8:** Vaccine hesitancy by region, sex, and age of respondents at the time of the vaccine hesitancy survey in Ethiopia, 2022.

Variable	Category	Hesitancy		*p*-Value
		No (%)	Yes (%)	
Region	Addis Ababa	102 (40.30)	151 (59.7)	*p* < 0.001
	Amhara	78 (33.33)	156 (66.67)	
	Sidama	60 (52.17)	55 (47.82)	
	Oromia	112 (42.42)	152 (57.58)	
	Afar	41 (21.5)	150 (78.5)	
	Somali	90 (29.7)	213 (70.3)	
Gender	Male	237 (34.7)	446 (65.3)	*p* = 0.505
	Female	247 (35.6)	431 (64.4)	
Age	Youth	120 (37.9)	197 (62.1)	*p* = 0.33
	Adult	364 (34.9)	680 (65.1)	

**Table 9:** Study participants perceived community and individual benefits of COVID-19 vaccination at the time of the vaccine hesitancy survey, Ethiopia, 2022.

Variable	Category	Perceived Vaccine Importance to the Community		*p*-Value	Perceived Vaccine Importance to the Individual		*p*-Value
		No	Yes		No	Yes	
Region	Addis Ababa	90 (35.6)	163 (64.4)	*p* < 0.001	75 (29.6)	178 (70.4)	*p* < 0.01
	Amhara	84 (35.9)	150 (64.1)		96 (41.0)	138 (59)	
	Sidama	65 (56)	51 (44)		48 (41.4)	68 (58.6)	
	Oromia	124 (47)	140 (53)		81 (30.7)	183 (69.3)	
	Afar	103 (54)	88 (46)		110 (57.6)	81 (42.4)	
	Somali	158 (52.1)	145 (48.5)		157 (51.8)	146 (48.2)	
Gender	Male	306 (44.8)	377 (55.2)	*p* = 0.437	294 (43.0)	389 (57)	*p* = 0.298
	Female	318 (46.9)	360 (53.1)		273 (40.3)	405 (59.7)	
Age	Youth	138 (43.5)	179 (56.5)	*p* = 0.345	133 (42.0)	184 (58)	*p* = 0.903
	Adult	486 (46.5)	558 (53.5)		434 (41.6)	610 (68.4)	

## Data Availability

The data presented in this study are available on request from the corresponding author. The data are not publicly available due to privacy reasons.

## Supplementary Materials

The following supporting information can be downloaded at: https://www.mdpi.com/article/10.3390/vaccines11040774/s1, Table S1: Questionnaire—A Study on COVID-19 Vaccine Hesitancy.
