# Supplementary material for: COVID-19 Knowledge, Attitudes, and Vaccine Hesitancy in Ethiopia: A Community-Based Cross-Sectional Study
Source: Vaccines (Basel). 2023 Mar 31;11(4):774. doi: 10.3390/vaccines11040774 (PMC10140841; doi:10.3390/vaccines11040774)
Supplement: Supplementary file 1 [file vaccines-11-00774-s001.zip › vaccines-2106418-supplementary.pdf]

A Study on COVID 19 Vaccine Hesitancy

	**Section 1. Informed Consent**		
VHC_00	Hello. My name is ________________ , and I'm working with Amref study on covid 19 vaccine hesitancy. We are talking with community members/health care workers/HEWs to better understand vaccine hesitancy, and I would like to invite you to participate. Before we proceed, I would like to ask you a few questions to confirm your consent to participate. Your opinion will help all concerned bodies to improve covid 19 vaccination coverage. I would like to ask you a few questions about your knowledge, perceptions and vaccination experiences. I will also ask you a few questions about your age, marital status, religion, and education level. Your name will not be recorded on any forms, and the information you provide us will be kept confidential. We will not share your individual responses with any members of the health care staff at this site. The interview will last approximately 15 minutes, and we will go to a private place to conduct the interview. Your participation is voluntary, and whether or not you participate will not affect any future care you receive. Some of the questions I ask might make you uncomfortable please remember that you may refuse to answer any questions or stop the interview for any reason. If during or after the interview you would like emotional, clinical or any type of professional support, I will provide you with the contact information for someone that you can contact, if you so desire. You will not get any special benefit because of your participation.		
VHC_01	Do you agree to complete this interview?	☐ 1 Yes<br>☐ 2 No →	end

	**Section 2. General information**		
VHS_02	Region	1. Addis Ababa<br>2. Amhara<br>3. Oromia<br>4. Sidama	
VHS_03	City/Sub-city	1. Gulelie<br>2. Yeka<br>3. Bahir Dar<br>4. Debre Birhan<br>5. Adama<br>6. Zeway<br>7. Hawassa	
VHS_04	District name/kebele	________________	
VHS_05	Date of interview (GC)	________________	
VHS_06	Name of the data collector	________________	
	**Section 3. Socio-demographic characteristics**		
**No.**	**Item**	**Response Options**	**Skip Pattern**
VHS_07	Age of respondent in years	______years	

VHS_08	Sex of respondents	1. Male<br>2. Female	
VHS_09	Religious status of respondent	1. Orthodox<br>2. Muslim<br>3. Protestant<br>4. Catholic<br>5. Other (specify___)	
VHS_10	Educational status of respondent	1. Can't read and write<br>2. Read and write only<br>3. Primary school (1-8 grade)<br>4. Secondary school (9-12 grade)<br>5. Certificate and above	
VHS_11	Occupation of respondents	1. Merchant<br>2. Housewife<br>3. Government employed<br>4. Private sector employed<br>5. Student<br>6. No job<br>7. Farmer<br>8. Other (specify____)	
VHS_12	Marital status of respondents	1. Married<br>2. Single<br>3. Divorced<br>4. Widowed	
VHS_13	If the respondent is married, educational status of partner	1. Can't read and write<br>2. Read and write only<br>3. Primary school (1-8 grade)<br>4. Secondary school (9-12 grade)<br>5. Certificate and above	
VHS_14	If the respondent is married occupation of partner	1. Merchant<br>2. House wife<br>3. Government employed<br>4. Private sector employed<br>5. Student<br>6. No job<br>7. Farmer<br>8. Other (specify____)	
VHS_15	Family Size	_______in number	
VHS-16	Average family income (Monthly)	-------------------------	
VHS-17	Average monthly expenditure (Monthly)	-------------------------	

	**Section 4. Wealth status indicator**		
**No.**	**Item**	**Response Options**	**Skip Pattern**
VHS_18	Does your household have electricity?	1. Yes<br>2. No	
VHS_19	Does your household have radio?	1. Yes<br>2. No	
VHS_20	Does your household have television?	1. Yes<br>2. No	
VHS_21	Does your household have a refrigerator??	1. Yes	

		2. No	
VHS_22	Does your household have electric mitad?	1. Yes<br>2. No	
VHS_23	Does your household have table?	1. Yes<br>2. No	
VHS_24	Does your household have chair?	1. Yes<br>2. No	
VHS_25	Does your household have bed with cotton?	1. Yes<br>2. No	
VHS_26	Does any member of this household have a bank account?	1. Yes<br>2. No	
VHS_27	What is the main source of drinking water for members of your household?	1. Piped to yard / plot<br>2. Other	
VHS_28	What kind of toilet facility do members of your household usually use?	1. Flushed latrine/Pit latrine without slab / open pit<br>2. No facility / bush / field<br>3. Other specify: ………….	
VHS_29	What type of fuel does your household mainly use for cooking?	1. Electric city<br>2. Wood/Charcoal<br>3. Other specify: ________	
VHS_30	What is the main material of the floor in your household?	1. Earth/sand<br>2. Other	
VHS_31	What is the main material of the exterior walls in your household?	1. Mud<br>2. Cement<br>3. Other specify:	
VHS_32	What is the main material of the roof in your household?	1. Metal / corrugated iron<br>2. Other	
VHS_33	Does the household member have vehicle?	1. Yes<br>2. No	
VHS_34	Does the household member have motor bicycle?	1. Yes<br>2. No	
VHS_35	Does the household member have bicycle?	1. Yes<br>2. No	

Section 5: KAP assessment questions
Section 5.1: Knowledge on covid 19 prevention and control

**No.**	**Item**	1. **Response Options**	**Skip Pattern**
VHS_36	What are the sign and symptoms of Covid-19?	2. Cough / sneezing / congestion / sore throat<br>3. Fever<br>4. Headache<br>5. Shortness of breath and difficulty breathing / chest pain<br>6. Muscle aches and / or joint pain /general body weakness<br>7. Symptoms of GI (diarrhea, nausea, vomiting, abdominal pain)	

		8. Loss of smell and / or taste<br>9. Other specify............<br>10. I do not know	
VHS_37	Covid 19 may not also show symptoms	1. Yes<br>2. No	
VHS_38	What are Covid-19 transmission methods?	1. Direct contact with persons affected by Covid-19<br>2. Touching things<br>3. Airborne<br>4. Other specify ....<br>5. 5. I do not know	
VHS_39	Who is at risk for severe CVD-19 infection?	1. People aged 65 years of age and older<br>2. People with non-communicable diseases<br>3. People with known respiratory diseases<br>4. Other specify ....<br>5. 5. I do not know	
VHS_40	Covid 19 can be spread by an infected person without symptom	1. Yes<br>2. No	
VHS_41	Covid 19 can be transmitted by respiratory droplets of infected person	1. Yes<br>2. No	
VHS_42	Covid 19 can be spread by the dead bodies of the infected person	1. Yes<br>2. No	
VHS_43	Avoiding going to crowded places can be prevent contacting Covid 19	1. Yes<br>2. No	
VHS_44	Avoiding travelling across cities can prevent contracting Covid 19	1. Yes<br>2. No	
VHS_45	Isolation of infected persons can prevent contracting covid 19	1. Yes<br>2. No	
VHS_46	What are the methods you know to prevent covid 19?<br>NB: Multiple response is possible	1. Wash hands regularly using soap and water<br>2. Wearing masks<br>3. Physical distancing<br>4. Clean with sanitizer<br>5. Getting vaccination<br>6. Avoid close contact with anyone who has a fever and cough<br>7. Stay at home<br>8. Cover mouth and nose when coughing or sneezing<br>9. Avoid direct contact with surfaces<br>10. Cook meat and eggs well	
**Section 5.2: Attitude on covid 19 prevention and control**			
VHS_47	Handling covid 19 will be more difficult if people or other community not keeping up with the information related to prevention	1. Agree<br>2. Don't know<br>3. Disagree	

VHS_48	Handling covid 19 will be more difficult if people or other community no longer need to worry about contracting covid 19	1. Agree<br>2. Don't know<br>3. Disagree	
VHS_49	Handling covid 19 will be more difficult if people or other community influence be negative news	1. Agree<br>2. Don't know<br>3. Disagree	
VHS_50	I feel that the reason experiencing a symptom or persons infected should compliance the protocol such as wearing mask	1. Agree<br>2. Don't know<br>3. Disagree	
VHS_51	I feel that person experiencing the symptoms or persons infected should be motivated to increasing implement covid 19 prevention measures and ensuring a healthy life.	1. Agree<br>2. Don't know<br>3. Disagree	
VHS_52	I feel covid 19 is Government or media propaganda	1. Agree<br>2. Don't know<br>3. Disagree	
VHS_53	I feel covid 19 is a curse	1. Agree<br>2. Don't know<br>3. Disagree	
**Section 5.3: Practice on covid 19 prevention and control**			
VHS_54	Do you wear a mask in crowded or public places	1. Never<br>2. Some times<br>3. Often	
VHS_55	Do you keep distance between (physical distance) in crowded or public places	1. Never<br>2. Some times<br>3. Often	
VHS_56	Do you use hand sanitizer and take a bath after going to a crowded or public place	1. Never<br>2. Some times<br>3. Often	
VHS_57	Do you change your cloths after going to a crowded or public place	1. Never<br>2. Some times<br>3. Often	
VHS_58	Do you carry out a campaign to prevent the spread of covid 19 by providing a direct example in daily activity	1. Never<br>2. Some times<br>3. Often	
VHS_59	Do you eat fruits and vegetables in the last few days	1. Never<br>2. Some times<br>3. Often	
VHS_60	In the last few days, do you exercise routinely	1. Never<br>2. Some times<br>3. Often	

**Section 6. Vaccine hesitancy**			
**6.1. Vaccination status**			
**No.**	**Item**	**Response Options**	**Skip Pattern**
VHS_61	Have you ever tested for COVID 19?	1. Yes<br>2. No →	VHS_63
VHS_62	If yes for the above question, what was the test result?	1. Positive<br>2. Negative	

VHS_63	If no for the above question, why you never tested	1. I scared testing<br>2. Because I don't trust the test<br>3. No solution knowing it<br>4. I have never had Covid-19 symptoms<br>5. Others (specify (_______)	
VHS_64	Are you vaccinated against COVID-19 with any of the vaccines available?	1. Yes →<br>2. No →	→ 6.1.1<br>→ 6.1.2
**6.1.1.**	**For vaccinated person section**		
VHS_65	What was the reason to take the vaccine?	1. I am old<br>2. I have comorbidity<br>3. I am too vulnerable<br>4. Covid-19 vaccine campaign/promotion<br>5. Other (specify____)	
VHS_66	Which of the vaccines did you receive?	1. Oxford, Astra Zeneca<br>2. Johnson & Johnson<br>3. BioNTech, Pfizer<br>4. Moderna Abdala/Soberana (Cuban)<br>5. Can't recall	
VHS_67	Which dose you take?<br>***NB: Depending the type of vaccines ask the round***	1. First<br>2. Second<br>3. Third/Booster	
VHS_68	When you vaccinated the recent vaccine?	__________months ______years	
VHS_69	Where you vaccinated?	1. Health facility<br>2. Campaign site	
VHS_70	Did you feel any adverse effect/s?	1. Yes<br>2. No →	→ VHS_72
VHS_71	If yes, what was the feeling/s of adverse effect/s	1. Pain, redness or swelling at injection site<br>2. Fever<br>3. Fatigue<br>4. Headache<br>5. Muscle pain<br>6. Chills<br>7. Joint pain<br>8. Nausea and vomiting<br>9. Swollen lymph nodes<br>10. Feeling unwell<br>11. Other specify: ---------------------	
VHS_72	Did you choose your vaccination type, or did you just take what was available at the time?	1. 1.Chose my preferred vaccine<br>2. Took what was available	
VHS_73	When you took the vaccine, were u given the detail information about the vaccines- type, possible immediate adverse effect…?	1. Yes<br>2. Yes, but not satisfied as the information was not detail<br>3. No	
VHS_74	If you choose- was your choice of vaccination based on medical advice or your own enquiry/investigation?<br><br>***NB: multiple response is possible***	1. Personal enquiry/investigation<br>2. Medical advice<br>3. Other (specify….)	

VHS_75	Were you initially hesitant to take the vaccine?	1. Yes, I was totally hesitant<br>2. Yes, to some extent I was hesitant<br>3. No →	VHS_77
VHS_76	If you were hesitant, please explain why you were initially hesitant?<br><br>***NB: multiple response is possible***	1. Not relevant, I was never hesitant<br>2. I did not qualify to receive it before<br>3. I did not like any of the vaccine options available to me<br>4. I had religious grounds for not taking it<br>5. I had a medical condition that barred me from taking it<br>6. I did not trust the government<br>7. I did not trust medical authorities<br>8. I did not trust the vaccines (not safe, developed too quickly, do not know what is in it)<br>9. I was not sure about long-term side effects of the vaccine<br>10. There were bad talks in the community about the vaccination and I was afraid<br>11. Worrying because of vaccine politics	
VHS_77	If you were hesitant, what changed your mind? (Select option that best applies).<br><br>***NB: multiple response is possible***	1. Not relevant I was never hesitant<br>2. I was offered a vaccine I was more comfortable with<br>3. I spoke to my doctor<br>4. I did more search<br>5. I consulted family and friends<br>6. My spiritual/religious leader advised me do it<br>7. I was persuaded by government/public health authorities<br>8. I noticed that a personality (public or otherwise) took it<br>9. I was forced by my employer/family members<br>10. I did not have a choice<br>11. I felt more comfortable because several people took it before me<br>12. I was frightened by the increased infections/deaths<br>13. My circumstances changed (such as being pregnant or any other medical or personal factor)<br>14. Other specify: -----------------------------------	
VHS_78	Why do you think the main reason for the community or the people who did not take vaccine?<br><br>***NB: multiple response is possible***	1. Do not know the vaccine<br>2. Lack of access for the vaccine<br>3. The hesitance/trust about the vaccine<br>4. Many people scared of Side-Effects of the Vaccine<br>5. other specify: ----------------------	
VHS_79	Do you advise all other eligible persons (aged from 18 and above) others to be vaccinated?	1. Yes<br>2. No; I don't	
6.1.2.	**People who have not been vaccinated**		
**No.**	**Item**	**Response Options**	**Skip Pattern**

VHS_80	Which of these reasons best explains why you are unvaccinated at this time or before?<br><br>***NB: multiple response is possible***	1. No access to take vaccine/ I cannot easily find the vaccine<br>2. I did not qualify to receive it<br>3. Can't find the time to go<br>4. Vaccine delivery sites are too distant<br>5. I don't like any of the vaccine options available to me<br>6. I will not take it on religious grounds<br>7. I believe that I have a medical condition that bars me from taking it<br>8. I do not trust the government/medical authorities here<br>9. I do not trust the vaccine (not safe, developed too quickly, do not know what is in it)<br>10. I believe it is a choice and I choose not to vaccinate with no reason<br>11. I won't take it<br>12. I am doubtful about long-term side effects of the vaccine<br>13. It is not mandatory for my work<br>14. No need to take since it will not protect<br>15. Other specify -------------------------------	
VHS_81	Have you a plan to be vaccinated?	1. Yes<br>2. No →	VHS_83
VHS_82	If yes, which vaccine do you prefer?	1. Oxford, AstraZeneca<br>2. Johnson & Johnson<br>3. BioNTech, Pfizer<br>4. Moderna Abdala/Soberana (Cuban)<br>5. Any as I do not have any detail information<br>6. Any as all vaccines work	
VHS_83	If "No" for VHS_80, why you never tested?	1. I scared testing before the vaccine<br>2. I have never had Covid-19 symptoms<br>3. I was sick for COVID 19 and I will never be reinfected<br>4. Others specify: …………….	
VHS_84	Has your view on the COVID-19 vaccination always been the same or has it changed over time?	1. No change- I still am not going to take it<br>2. I am now more inclined towards taking it<br>3. I am now less inclined towards taking it	
VHS_85	Why do you think the main reason for the community did not taking vaccine?	1. Do not know the vaccine<br>2. Lack of access for the vaccine<br>3. The hesitance/trust about the vaccine<br>4. Many people scared of Side-Effects of the Vaccine<br>5. other specify: ----------------------	
VHS_86	Would persuade you to change your mind and take the COVID-19 Vaccine because of?<br><br>***NB: multiple response is possible***	1. If it were necessary for me to secure or maintain a job<br>2. If it would allow me to access social activities more freely<br>3. If I was given more scientific or medical information	

		4. If I saw people, I care about getting sick/dying from COVID-19<br>5. If I saw influential people who now oppose it switch their position<br>6. If it was required for me to travel overseas<br>7. Other reason (Specify) ________________	
VHS_87	If you were to choose between expanding the level of vaccination in the country and prolonged restrictions, which would you prefer?	1. Increase vaccination levels<br>2. Continue restrictions	
VHS_88	Apart from vaccinations, what do you think of these other options for combating COVID-19:	1. No need for any options, COVID-19 is a hoax<br>2. Natural immunity (let the vulnerable get sick and the rest of us get on with life)<br>3. Comprehensive lockdowns<br>4. More hand washing<br>5. Regular face mask use<br>6. Better social distancing<br>7. Other specify ---------------------	
VHS_89	For who would you support the implementation of COVID-19 vaccine mandates?<br><br>***NB: multiple response is possible***	1. New visitors<br>2. Frontline medical/Elder care workers<br>3. Public servants<br>4. Workers in the Hotel/Tourism sector<br>5. Taxi/Minibus drivers and conductors<br>6. Secondary/Tertiary school teachers and students<br>7. Other (specify-----------------------------)	
**Section 7: Primary and Trusted Source of information**			
VHS_90	What is your PRIMARY source of information on the COVID-19 vaccine situation?<br><br>***NB: multiple response is possible***	1. Government/Official sources including HCWs, HEWs<br>2. Printed/posted materials- leaflet, billboard,<br>3. Private/personal medical sources<br>4. Social media (Facebook/Instagram/WhatsApp/Twitter/TikTok/YouTube)<br>5. Personal internet research (via Google, Safari, Bing, Yahoo, Baidu, AOL, Ask.com, Excite)<br>6. Local radio/television/newspapers<br>7. Information from family and friends<br>8. Religious leaders	
VHS_91	Specifically, what type of information on the COVID-19 Vaccine would help you to be more inclined to take it?<br><br>***NB: multiple response is possible***	1. The side effects of the vaccine<br>2. How much the vaccine is effective?<br>3. The numbers of people who got sick/died and their vaccination status<br>4. The different types of available vaccines<br>5. Location of vaccination sites<br>6. The attitude of my religion or religious leaders<br>7. Getting clear information about the misconceptions you hear about the vaccine<br>8. The impact of the vaccine on my sexual health<br>9. The impact of the vaccine on my ability to have children	

		10. Other information (specify)	
VHS_92	Have you ever consulted a medical advice?	1. Yes, I consulted<br>2. No, I did not consult<br>3. Medical advice was irrelevant in my case	
VHS_93	What is your TURSTED source of information on the COVID-19 vaccine situation?<br><br>***NB: multiple response is possible***	1. Government/Official sources including HCWs, HEWs<br>2. Printed/posted materials- leaflet, billboard,<br>3. Private/personal medical sources<br>4. Social media (Facebook/Instagram/WhatsApp/Twitter/TikTok/YouTube)<br>5. Personal internet research (via Google, Safari, Bing, Yahoo, Baidu, AOL, Ask.com, Excite)<br>6. Local radio/television/newspapers<br>7. Information from family and friends<br>8. Religious leaders<br>9. Other (specify____)	
VHS_94	What is the best way to communicate with you or the way that you generally prefer to receive information?<br><br>***NB: multiple response is possible***	1. Via newspapers (paper/electronic)<br>2. Via radio<br>3. Via television (local or cable)<br>4. Via Facebook<br>5. Via Instagram<br>6. Via WhatsApp<br>7. Via Twitter<br>8. Via TikTok<br>9. Via YouTube<br>10. Social networks (family and friends)<br>11. Other (specify______)	
VHS_95	What is the best day to get in touch with you or to receive information?<br>***NB: multiple response is possible***	1. Monday<br>2. Tuesday<br>3. Wednesday<br>4. Thursday<br>5. Friday<br>6. Saturday<br>7. Sunday	
VHS_96	What time you usually prefer to get information in a typical day?<br><br>***NB: multiple response is possible***	1. Morning<br>2. Mid-day- during lunch<br>3. Afternoon<br>4. Late evening	

**Section 9. actors for covid 19 vaccine hesitancy**			
i. Perceived susceptibility to covid -19			
No	Item	Response Options	Skip Pattern
VHS_97	It is likely that I will get COVID-19	1. Strongly disagree<br>2. Disagree<br>3. Neutral<br>4. Agree<br>5. Strongly agree	
VHS_98	I am at risk of getting COVID-19	1. Strongly disagree<br>2. Disagree	

		3. Neutral<br>4. Agree<br>5. Strongly agree	
VHS_99	It is possible that I will get COVID-19.	1. Strongly disagree<br>2. Disagree<br>3. Neutral<br>4. Agree<br>5. Strongly agree	
VHS_100	I think I infected by Covid-19 and get cured though I am not formally diagnosed/tested	1. Strongly disagree<br>2. Disagree<br>3. Neutral<br>4. Agree<br>5. Strongly agree	
**ii. Perceived severity of COVID-19**			
VHS_101	I believe that COVID-19 is a severe health problem	1. Strongly disagree<br>2. Disagree<br>3. Neutral<br>4. Agree<br>5. Strongly agree	
VHS_102	I Believe that COVID-19 has serious negative consequences.	1. Strongly disagree<br>2. Disagree<br>3. Neutral<br>4. Agree<br>5. Strongly agree	
VHS_103	I believe that COVID-19 is extremely harmful.	1. Strongly disagree<br>2. Disagree<br>3. Neutral<br>4. Agree<br>5. Strongly agree	
iii. ***Perceived individual benefits of COVID-19 vaccines***			
VHS_104	COVID-19 vaccines will work in preventing the disease	1. Strongly disagree<br>2. Disagree<br>3. Neutral<br>4. Agree<br>5. Strongly agree	
VHS_105	If I get the vaccines, I will be less likely to get severe form of COVID-19	1. Strongly disagree<br>2. Disagree<br>3. Neutral<br>4. Agree<br>5. Strongly agree	
VHS_106	If I get the vaccines, I will be less likely to get COVID-19 disease	1.Strongly disagree<br>2.Disagree<br>3.Neutral<br>4.Agree<br>5.Strongly agree	
iv. ***Perceived community benefits of COVID-19 vaccines***			

VHS_107	Having myself vaccinated against COVID-19 is beneficial for the health of others in the community?	1.Strongly disagree<br>2.Disagree<br>3.Neutral<br>4.Agree<br>5.Strongly agree	
VHS_108	COVID-19 vaccines protect the health of the community?	1.Strongly disagree<br>2.Disagree<br>3.Neutral<br>4.Agree<br>5.Strongly agree	
v.	**Perceived barriers of getting COVID-19 vaccines (safety and cost concerns).** How much would the following factors prevent you from getting vaccinated for COVID-19		
VHS_109	Concerns about whether COVID-19 vaccines are safe	1. Strongly disagree<br>2. Disagree<br>3. Neutral<br>4. Agree<br>5. Strongly agree	
VHS_110	Not enough research done on COVID-19 vaccines	1. Strongly disagree<br>2. Disagree<br>3. Neutral<br>4. Agree<br>5. Strongly agree	
VHS_111	I have concerns about possible side effects of COVID-19 vaccines	1. Strongly disagree<br>2. Disagree<br>3. Neutral<br>4. Agree<br>5. Strongly agree	
**vi.**	**Self-efficacy about covid 19 vaccine**		
VHS_112	I will be able to take the vaccine to prevent transmitting COVID-19 to others	1.Strongly disagree<br>2.Disagree<br>3.Neutral<br>4.Agree<br>5.Strongly agree	
VHS_113	It will be easy for me to get the vaccines to protect myself from COVID-19	1.Strongly disagree<br>2.Disagree<br>3.Neutral<br>4.Agree<br>5.Strongly agree	
VHS_114	Getting vaccinated to prevent COVID-19 is convenient	1.Strongly disagree<br>2.Disagree<br>3.Neutral<br>4.Agree<br>5.Strongly agree	
**vii.**	***Cues to action (COVID-19 exposure)***		
VHS_115	Have you experienced any of the following in the past 9 months?	1. Tested positive for COVID-19 antibody	

	NB: multiple response is possible	2. I was hospitalized because of Covider-19<br>3. Family or close friend tested positive for COVID-19<br>4. Someone you knew tested positive for COVID-19<br>5. Exposed to people who have got COVID-19<br>6. Treating patients with COVID-19<br>7. Taking care of someone with COVID-19<br>8. Family or close friend passed away due to COVID-19<br>9. Someone you knew passed away due to COVID-19<br>10. None of above	
VHS_116	Were you an essential worker during the COVID-19 lockdown?	1 Yes<br>2 No →	*VHS_120*
VHS_117	If your response is YES for VHS_116, most people who are like me will get COVID-19 vaccination.	1. Strongly disagree<br>2. Disagree<br>3. Neutral<br>4. Agree<br>5. Strongly agree	
VHS_118	If your response is YES for VHS_116, Most people who are important to me will get COVID-19 vaccination	1. Strongly disagree<br>2. Disagree<br>3. Neutral<br>4. Agree<br>5. Strongly agree	
VHS_119	If your response is YES for VHS_116, Most people who are important to me think that I should get COVID-19 vaccine	1. Strongly disagree<br>2. Disagree<br>3. Neutral<br>4. Agree<br>5. Strongly agree	
VHS_120	How would you feel if you were vaccinated with Covid-19 vaccine?	1. Negative<br>2. Positive	
viii. Experience with*past* vaccination			
VHS_121	Have you ever not accepted covid 19 vaccination?	1. Yes<br>2. No	
VHS_122	Most people tolerate covid 19 vaccination very well	1. Strongly disagree<br>2. Disagree<br>3. Neutral<br>4. Agree<br>5. Strongly agree	
VHS_123	Do you know someone who had a bad reaction due to covid 19 vaccinations and that made you not to vaccinate?	1. Yes<br>2. No	
VHS_124	Do you encounter someone with a serious complication or someone who is died because of covid 19 vaccination?	1. Yes<br>2. No	

VHS_125	Do you know someone who encountered serious complication or died because of covid 19 disease?	1. Yes<br>2. No	
VHS_126	If yes to VHS_124, did this make you to reconsider your decision to get vaccinated?	1. Yes<br>2. No	
VHS_127	Geographical location (GPS)	____________________	
